# The development of the internal medicine courses at Hannover Medical School from 2001 to 2018

**DOI:** 10.3205/zma001264

**Published:** 2019-10-15

**Authors:** Philip Bintaro, Sabine Schneidewind, Volkhard Fischer

**Affiliations:** 1 Medizinische Hochschule Hannover, Klinik für Nieren- und Hochdruckerkrankungen, Hannover, Germany; 2 Medizinische Hochschule Hannover, Klinik für Gastroenterologie, Hepatologie und Endokrinologie, Hannover, Germany; 3 Medizinische Hochschule Hannover, Studiendekanat, Bereich Evaluation und Kapazität, OE 9135, Hannover, Germany

**Keywords:** internal medicine, curriculum development, program structure, study success, medicine

## Abstract

**Aim: **The subject-based model curriculum at the Hannover Medical School (MHH) is characterized by two major features: early and continuous contact with patients and the interconnection of theoretical and clinical content. The progressive adaptations to the internal medicine curriculum which is taught longitudinally over five years are presented.

**Methods: **Looking back on 17 years of experience with our model curriculum and the events leading to its inception, we summarize the challenges and subsequent modifications to the longitudinal internal medicine curriculum. Some of these challenges are likely to occur in other subjects and can therefore be generalized.

**Results: **Integrating preclinical and clinical content was more resource intensive than thought and unexpectedly led to capacity problems since the German courts ruled that the presence of more teachers brought with it an obligation to enroll more students. In response to this, teaching responsibilities were extended to both outpatient facilities and academic teaching hospitals. Major changes included a more even distribution of clinical content in the first year, a rigorous standardization of teaching content in clinical skills, increased adaptation of content to reflect prior knowledge in the third and fourth years, and a focus on clinical reasoning in the fifth year. Restructuring the academic year into three ten-week blocks (two in the winter term and one in the summer term) allowed the retention of small groups.

**Conclusion:** These measures rely heavily on considering teaching responsibilities within rotation plans, curriculum development and continuous faculty engagement. Since teachers are not sufficiently familiar with the distinctions between teaching formats, they mostly consider how far students have advanced in their studies when choosing course content. This implies that the current nomenclature requires improvement.

## Zusammenfassung

**Zielsetzung: **Ein wesentlicher Bestandteil des fachbezogenen Modellstudiengangs an der Medizinischen Hochschule Hannover sind die klinisch-praktischen Module, die sich an zwei Kriterien orientieren: Früher und kontinuierlicher Kontakt mit Patienten; wiederholte Verknüpfung von Theorie und Praxis. Deren Entwicklung in den letzten 17 Jahren wird einschließlich der Vorgeschichte dargestellt.

**Methode: **Im Rückblick auf die Erfahrungen mit dem Modellstudiengang und die Gründe für seine Etablierung werden die Herausforderungen und dadurch ausgelösten Veränderungen des longitudinalen Curriculums skizziert. Einige der Herausforderungen sind auch an anderen Standorten erwartbar und deshalb von allgemeinem Interesse.

**Ergebnisse:** Weil die Integration vorklinischer und klinischer Inhalte ressourcenintensiv, aber politisch gewünscht ist, waren die kapazitätsrechtlichen Probleme unerwartet. Insbesondere die Auffassung, dass mehr Dozierende automatisch mehr Ausbildungskapazität bedeuten, war nur schwer mit dem didaktischen Anspruch vereinbar. Um diesen aufrecht zu erhalten, wurden externe Krankenhäuser und niedergelassene Praxen vermehrt einbezogen. Die wichtigsten Veränderungen in den Jahren betrafen die Verteilung klinischer Lehrinhalte im ersten Studienjahr, eine rigorose Standardisierung der Ausbildung in Diagnostischen Methoden, eine verstärkte Berücksichtigung schon erworbener Fähigkeiten im Studienverlauf und die Fokussierung auf klinische Entscheidungsfindung im 5. Studienjahr. Die Restrukturierung des akademischen Jahres in drei zehnwöchige Tertiale (zwei im WS und eins im SS) garantierte die Beibehaltung kleiner Gruppen.

**Schlussfolgerung: **Die genannten Maßnahmen setzen eine gründliche Berücksichtigung von Lehrstrukturen und didaktischen Fähigkeiten voraus, um Rotationspläne, Curriculumsentwicklung und das Dozentenengagement nachhaltig aufeinander abzustimmen. Weil den Dozierenden die Unterschiede zwischen den Lehrformaten nicht hinreichend bekannt sind, orientieren sie ihre Lehrinhalte am Studienjahr, in dem sich die Studierenden befinden. Die aktuell verfügbare Nomenklatur ist deshalb weiterzuentwickeln.

## 1. Introduction

With the eighth revision of the German act licensure act of physicians (ÄAppO) [1], the strict requirements for medical education in Germany were relaxed to include the “model clause” that allowed medical schools four basic options to set up model curricula in addition to the conventional curriculum

1. The preliminary medical examination and the first part of the state medical examination were no longer fixed requirements.
2. The nursing traineeship and the clinical clerkships could be completed at a different point in time than that prescribed by the conventional curriculum.
3. The final practical year of medical study could be structured differently.
4. External hospitals and medical practices could be included in the instruction during all educational phases.

With the ninth revision of the licensure act of physicians (ÄAppO) [2], even more leeway was given to the medical schools beginning in the 2003/04 winter semester. Among all of the changes, three are of concern here. First, the first and second parts of the state medical examination were no longer required. At the same time, the medical schools were tasked with administering university-specific graded exams. Second, additional clinical clerkships were introduced for five subjects that, depending on the medical school, were to last between one and six weeks. The educational opportunities connected with this and the problems related to the rules and regulations regarding student selection have already been described elsewhere [3]. Third, the implicit mandate to continue a conventional study program in parallel to an established model curriculum no longer held.

Parallel to these national developments in the 2001/02 winter semester, Hannover Medical School (MHH) introduced for the first time rules regarding part-time study during the clinical phase [4]. The aim was to give students greater rights while also explicitly identifying several responsibilities. It is possible to view the passage of these new study regulations as the moment when the professionalization of university-level teaching began at MHH. Simultaneously, it is also evidence of the acceptance that simply bringing enthusiastic teachers and eager-to-learn students together in a campus setting no longer sufficed.

In particular, the rule that applied until the ninth revision of the ÄAppO and stated that the formal certificates of attendance (*Scheine*) for the both clinical study phases required only regular attendance but not successful participation had, on the one hand, given students extensive freedom, that at MHH led to the situation in which for many students the fifth year of study entailed no such certificates and allowed them the option to pursue individual chosen paths, such as doctoral degrees, study abroad, elective courses or voluntary clerkships. However, this rule also led to the consequence that many clinical lectures were given to nearly empty auditoriums. In addition to this, the use of bedside teaching made the weekly attendance certificates MHH students were used to obtaining in the preclinical phase unfeasible, which in turn extended the average length of study significantly beyond that of the conventional study program because external supervision was missing.

The long-standing link between the imparting the theoretical principles of medicine and practical clinical training [5], [6] threatened to fall apart between 2002 and 2005 due to restrictions imposed by student admissions [7]. It seemed prudent to expand the patient-based medical education that was mandated by law, but still viewed as inadequate, and to develop a five-year integrated curriculum [8], [9]. 

## 2. Moving from a conventional curriculum to a model curriculum

The new rules regarding part-time study were used in particular by the Center for Internal Medicine to restructure courses and teaching. This resulted in a major lecture course in the fourth year of study that was closely interlinked and coordinated with bedside teaching. This block composed of lecture, seminar and bedside teaching, which was taught all day for multiple weeks, quickly came to be referred to as a “Block Practicum”.

However, the examinations connected with the eighth revision of the licensure act of physicians (ÄAppO) in the form of the preliminary medical examination taken between the preclinical and clinical study phases and the first part of the state examination taken between the third and fourth years of study hindered the curricular unity of the anatomy seminars in the first year, the course on physical examinations in the third and the block practicum in internal medicine in the fourth, even though the latter two were usually offered only by the Center for Internal Medicine.

In the ninth version of the licensure act of physicians, lawmakers required medical schools to implement new teaching formats, such as seminars with clinical relevance and integrated seminars in the preclinical phase to incorporate clinical subjects. For the instructors at MHH this sounded very reminiscent of the reformatory aims connected with the university’s founding and early days and they understood it as a call to meet this challenge anew [6]. It was conveniently forgotten that the earlier reform efforts meant for annual cohorts of 160 medical students would come to fail due to legal issues surrounding the number of additional new admissions.

In contrast to most other German medical schools, MHH pursued the integrational model in its organizational structure with close institutional links between the medical school and hospital. At the same time, MHH is one of the most active medical research institutions.

Given this context, MHH consciously chose a modular program in which the responsibility for individual subjects remained visible and the special attributes of the previous curriculum were retained under new conditions: appropriate inclusion of clinical content as early as possible, efficient preparation of the majority of student cohorts for the state examinations, and (for the most part) no required attendance certificates in the final semester. What emerged from this in 2003 was the HannibaL model curriculum, with the first two years still reflecting a conventional study program [10].

The long tradition at MHH of supplementing preclinical lectures with lectures by clinicians and the presentation of patients was expanded to include instruction by clinical practitioners in seminars also. This addition to the seminars was presented and described to students and the administrative law courts as a well-rounded educational concept.

**Figure 1:**
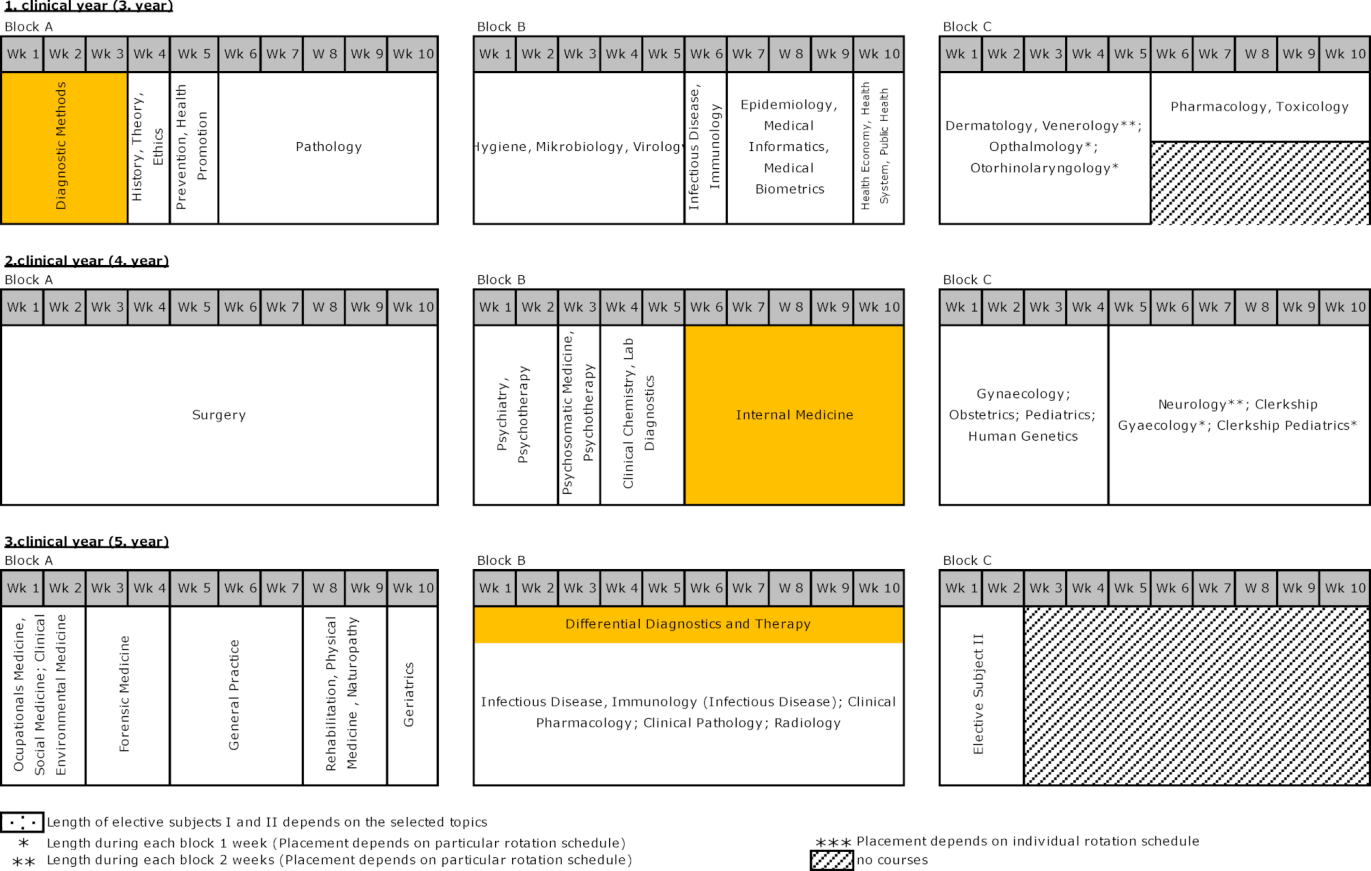
Overview of the HannibaL model curriculum 2003/04. Modules that are offered by the Center for Internal Medicine are shown in orange. The courses are offered three times per year and rotate during each year.

The actual changes, however, affected the third through fifth study years. The clinical instruction in block form during the fourth year in internal medicine, which was very well received by students, was then applied to all subjects in the third through fifth years of study. The resulting rotations had three blocks of ten-week courses, of which two blocks were offered in the winter semester and one in the summer semester. The student cohort was divided into three groups which then cycled through the three blocks by rotating each semester [7]. The ensuing course schedule that started in the 2003/04 winter semester is presented in figure 1 (Fig. 1). The modules that were designed partially or entirely by the Center for Internal Medicine are highlighted in orange so that the courses in internal medicine are easily visible.

**Figure 2:**
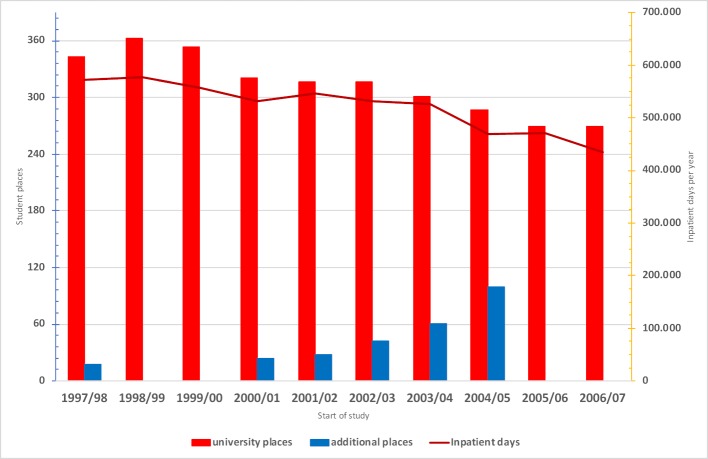
Development of student places and inpatient days for the previous year between 1997/98 and 2006/07 at MHH.

Although it was possible to spread the internal medicine curriculum more coherently over the years and thus align it with the students’ growing knowledge, a major disadvantage of the conventional curriculum remained present in the form of the first part of the medical state examination. In addition, there was an unexpected development. Contrary to what was promised by lawmakers, the administrative courts did not accept the premise that an increase in teaching load accompanied the new course formats in the preclinical and clinical phases, but rather interpreted the inclusion of clinical practitioners in the teaching of preclinical courses as an expansion of the university’s capacity to admit more students to the medical degree program. For this reason, MHH was required to create additional places for the first two years of study. However, the capacity to provide patient-based instruction sank rapidly since, as ordered by the German Council of Science and Humanities (Wissenschaftsrat), MHH had already begun to eliminate hospital beds. It quickly became clear that there was a real danger that the additional students who had successfully passed the preliminary medical examination could be forcibly disenrolled. In the academic year 2004/05 there was one additional place for every three regular places. Given the usual pass/fail rates it was foreseeable that ensuring graduation for at least half of the additional places at MHH could become impossible. Figure 2 (Fig. 2) illustrates this debilitating development that affected medical education at MHH.

Since MHH did not want to step back in any way from the educational improvements contained in its 2003 study regulations and increasingly saw the improvements as not going far enough, the creation of a model medical curriculum was explored in talks with Lower Saxony’s Ministry of Science and the Ministry of Social Affairs in the winter of 2004-2005. The model curriculum that was then launched in the 2005/06 winter semester had a series of changes regarding internal medicine compared to the conventional study program; these differences were basically seen in the improved practice-based instruction [11]. Marked in orange again, the internal medicine modules now began clinical education in the second week of the first year and continued until the sixth year of study. The elimination of the first part of the medical state examination in favor of an equivalent assessment spread out over two years contributed to a situation in which students who experienced initial difficulties were not necessarily faced with prolonged study time [12]. Above all, the clinical instruction given in the fourth year took on a higher quality as a result of these two aspects. Much more than the quantitative increase in bedside teaching and patient contact, that certainly was part of the reforms, the Center for Internal Medicine was much more invested in establishing a different kind of teaching and learning [13]:

- During the first semester, patient-centered medical practice connected with the preclinical and clinical theory pertaining to a larger topic (hypertension, back pain, (breast) cancer, pulmonary disease) was to be demonstrated and imparted to students within the hospital visitations that took place as part of the preparatory course.
- In the second half of the second year, the traditional course on conducting physical examinations was offered instead as a multidisciplinary course, but with clear focus on internal medicine, to prepare students for clinical electives. Close coordination with the Physiology module was specifically meant to foster clinical practice skills, underscoring the importance of medical theory to medical practice far beyond the learning assessment at the end of the module.
- During the third year of study, prior knowledge regarding the theory and practice of medicine was deepened using selected clinical pictures as part of an interdisciplinary lecture course (Clinical Medicine I) so that an authentic presentation of what physicians do in the course of their work could be used as a means to impart knowledge.
- Third-year students were also able to apply the knowledge and skills gained in the Diagnostic Methods module for the first time at the nearby teaching hospital. For three weeks students recorded case histories and conducted physical examinations for real hospital patients under the supervision of the physicians there. As is the case in the Diagnostic Methods module, a uniform check sheet was used to ensure that all students, regardless of which internal medicine ward they were assigned to, learned and applied the same framework of knowledge and skills.
- The Block Practicum in Internal Medicine was continued in the fourth year with the second part at the MHH Center for Internal Medicine. This existed alongside a lecture series consisting mostly of bedside teaching. Students were divided into groups of eight and assigned to three internal medicine clinics so that under appropriate supervision they could learn to diagnose and treat the broadest possible range of diseases.
- The culmination of this phase of study was the module on Differential Diagnostics and Therapy that was taken in the fifth year. The idea to implement the ten individual weeks, each one covering a major symptom in the form of object-related study groups was abandoned in favor of an interlinked lecture block that included the Clinical Pathology Conference and Clinical Pharmacology modules.

**Figure 3:**
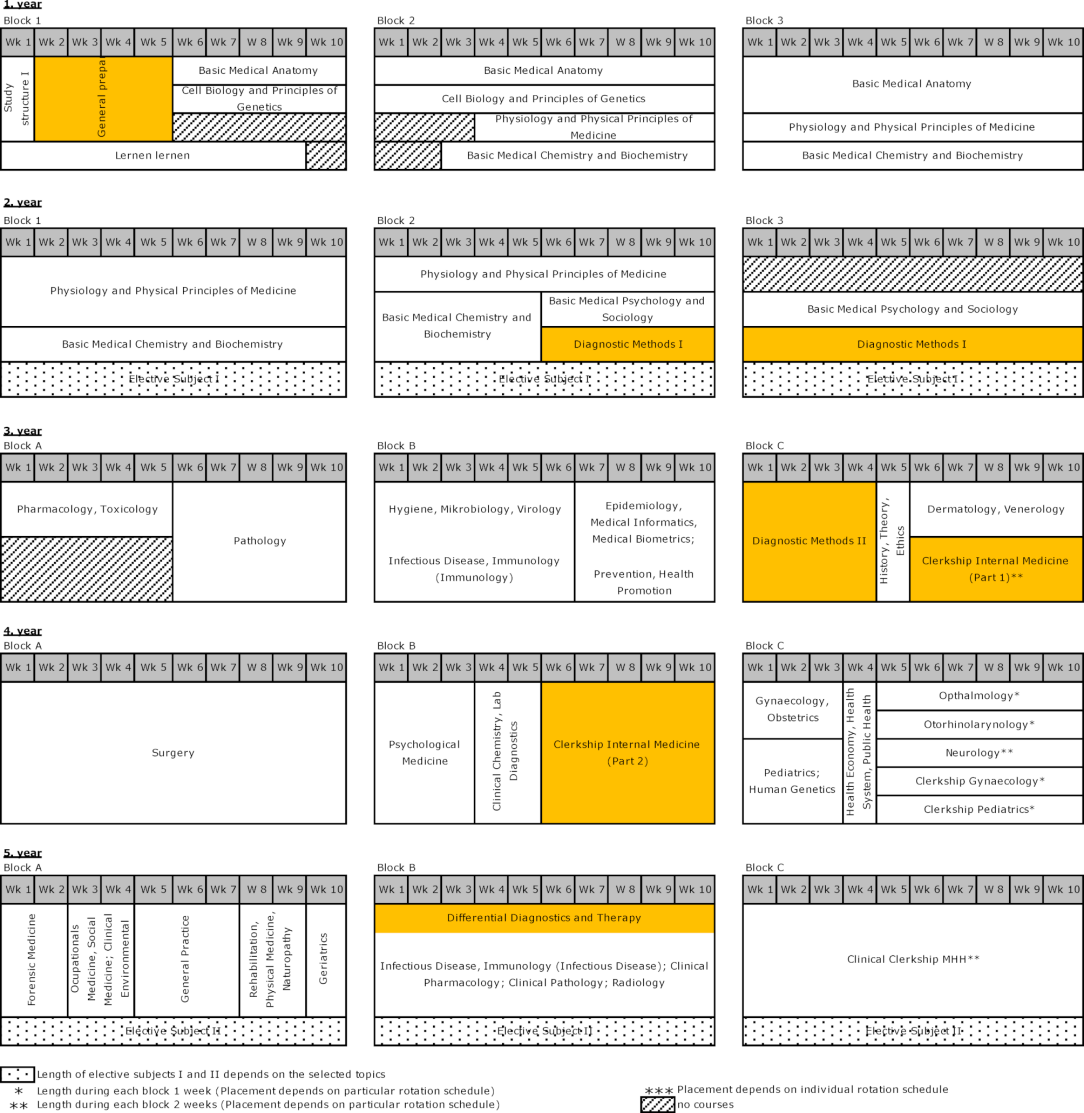
Overview of the HannibaL model curriculum 2005/06. The courses in years 3-5 are offered three times per year and rotate during each year.

Figure 3 (Fig. 3) shows the distribution of the modules over the first five years of study. It must be noted that the three teaching blocks in the first and second years of study are taught in the sequence shown, while the teaching blocks in years 3-5 are offered in constant rotation [7].

Finally, the internal medicine curriculum concluded in the final year of medical studies with the required trimester dedicated to Internal Medicine. However, we are not focusing on this or the development of the MHH log books, in which the curricular content is explicitly stated and came to represent a national approach.

## 3. Continued Development of the Reform

The *HannibaL* model curriculum intended to remedy weaknesses as quickly as possible and, with consideration for the regular student evaluations, modify the schedule overall and even target individual modules [9], [14]. It was only logical that the study rules and regulations have undergone small changes each year since 2009 after the initial phase of the model curriculum. The following now describes the current situation regarding development:

- The original four-week block for the preparatory course was transformed into one two-week and two one-week blocks each at the start of the teaching blocks in the first year of study. The continual learning assessment each week has not changed. With stronger distribution over the academic year, it was possible to adapt specific curricular content to match students’ progress in the Basic Anatomy module. The clinical visitations were largely kept as they had been.
- In the Diagnostic Methods module, developments concentrated on uniformly teaching and preparing all 55 student groups for the exam. This included the drafting of guidelines with course learning objectives, teacher training and the creation of interdisciplinary instructional videos [15]. Basic skills in radiology involving the interpretation of chest x-rays were integrated into the course as preparation for clinical electives later on. The final OSCE at the end of the module, whose overarching learning objectives include correct physical examination techniques and empathetic doctor/patient interaction, was extensively revised and developed.
- For the Clinical Medicine I module, the content of individual lectures needed to be re-coordinated because clear departures from the original concept had quietly taken hold over the years [16]. In addition, special value was placed on the adaptability of the module content to reflect prior knowledge accrued over the course of study [17].
- The first part of the Internal Medicine Block Practicum was initially taught at all of MHH’s teaching hospitals in form of a clinical clerkship. Due to mediocre evaluations, it has been reduced to a smaller number of nearby institutions. Requiring students to turn in 15 case histories and physical examination sheets as part of the curriculum proved inefficient and has been replaced by a structured oral patient presentation featuring immediate feedback.
- In the second part of the module during the fourth year of study, the bedside teaching has been replaced by clinical clerkships. The aim is to offer each student the opportunity to examine patients individually and to better integrate the students into the daily routine of the clerkship’s instructors. To ensure the required number of patients, substantially more outpatient clinics have been included in the program. Teacher training has been implemented in this context. Room for a seminar was created to enable deeper study of practical topics (e.g. emergency sonography). A non-standardized oral assessment has been eliminated. The unanimously desired introduction of a practical assessment format failed due to lack of necessary staff. An alternative format is being worked on.
- Analogous to the Clinical Medicine I module, Clinical Medicine II is undergoing a return to its original aim. All of the instructors have committed themselves to pay stronger attention to the connection between differential diagnoses and the major symptoms covered in the curriculum, as well as to use case-based and interactive teaching formats more frequently in place of the more traditional lecture format.

**Figure 4:**
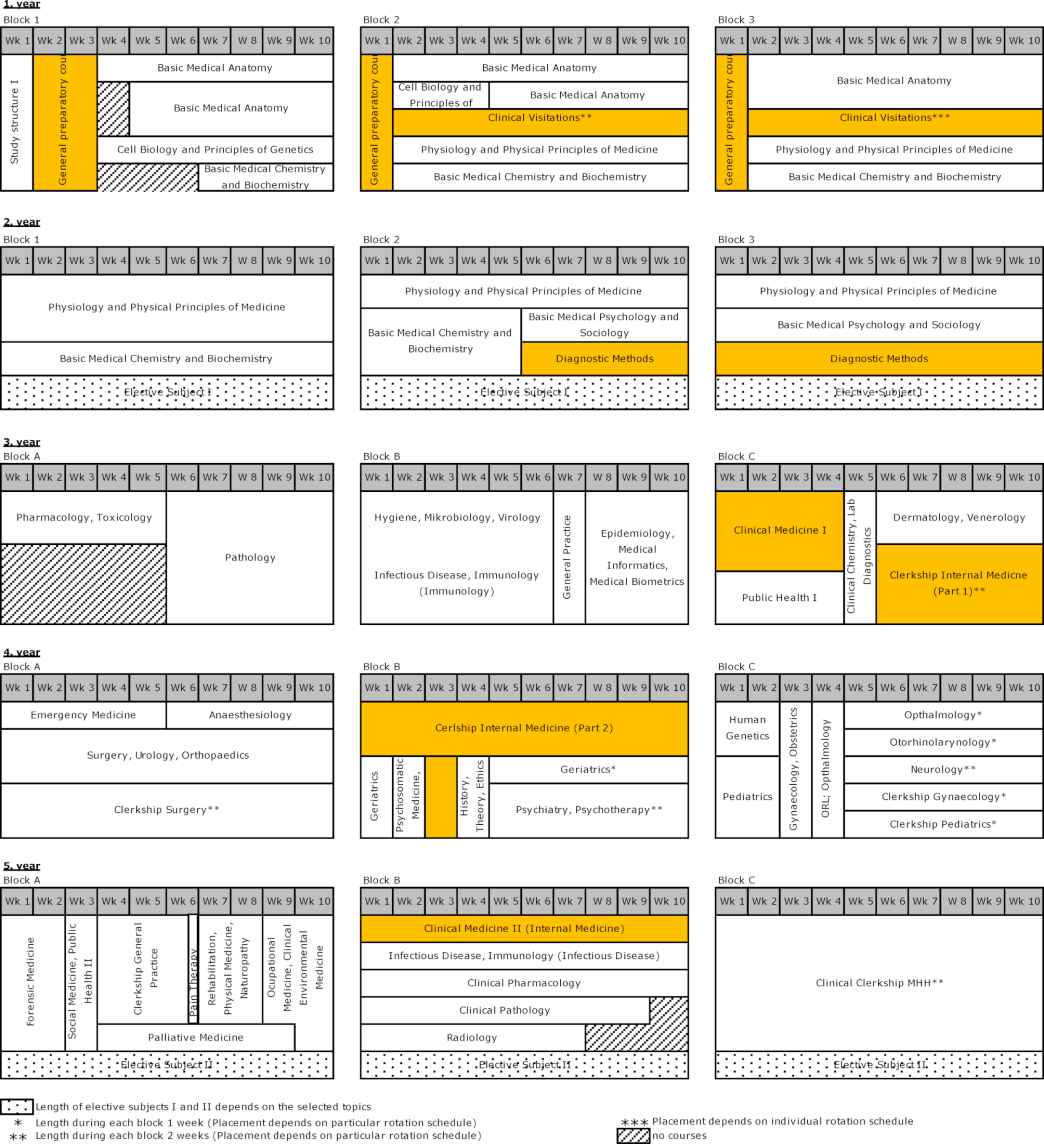
Overview of the HannibaL model curriculum 2017/18. The courses in years 3-5 are offered three times per year and rotate during each year.

The module’s sequencing over the five years of study, as presented in figure 4 (Fig. 4), shows these changes to the study program. Overall, it has been possible to spread the courses out more evenly over the calendar year in each year of study.

The log book for internal medicine at MHH is strongly based on the sample log book put forth by the Medinizinsche Fakultätentag (MFT). Explicit links to the curricular content of the internal medicine modules during the first five years of study has not yet been put into place for this log book.

## 4. Discussion

*HannibaL* was developed pragmatically based on existing teaching strategies. Its development did not follow a theoretical framework but did consciously embrace opposing principles [8], [9]. In parallel, new teaching formats, the reassessment of curricular content and teacher training were pushed forward in the internal medicine modules. The related problems were known in general, but foreseeable only in individual cases. It is evident from discussions with many instructors that the pedagogical definitions for various teaching formats [18][ are practically unknown. It is clearly the students’ level of study, and not the official teaching format, that determines the content imparted by the (medical) teachers: in the first year the focus is mainly on professional attitudes and interacting with patients; in the second year on the basic skills of taking case histories and conducting exams; and in the fourth and fifth years on differential diagnostics and clinical reasoning. It is against this background that the analysis of the model curriculum’s strengths, weaknesses and potential for development by Paulmann, Fischer & Just [7] must be viewed. We focus here on individual aspects that could be expanded upon in later studies since from the perspective of the instructors [19] and students [20] this educational approach has proven worthwhile, even if there remains a need for modifications.

While the subject-based modularization of the curriculum means that students concentrate intensively on a single subject, it is also the case that teachers and students are prevented from seeing the ways in which the medical disciplines and other subject are interlinked with each other because the priority has remained on optimizing the modules. The modules that had been integrated into the curriculum in an interdisciplinary manner were created usually at the behest of the Center for Internal Medicine. Contrary to expectations when the curriculum was implemented, there was neither an increase in the number of interdisciplinary courses within individual modules, nor could the interlinking of the modules in terms of a learning spiral be advanced as was intended. This process cannot even be considered finished for the modules presented here, for which the Center for Internal Medicine carries responsibility or at least is generally involved in their design. Still, these modules are now seen, not only by the instructors, but also by students, who for years equated internal medicine only with the fourth-year module, as having a perceptible sequence in terms of content.

For the instructors the reason for this lies in the still imperfect union between teaching, research and medical practice in the careers of individual instructors. This is not surprising when it is already challenging in the purely theoretical subjects to equally meet the demands for excellence in teaching and research. The drive toward excellence and budget constraints make it even more complex. For students, the learning assessment that functions as an equivalent to the first medical state examination and is spread out over the first two years of study may potentially narrow their perception of the curriculum’s overall coherence. Like the many responsibilities juggled by instructors, the sheer amount of learning material clearly hinders students from strategically planning their studies and finding an individual concentration at the beginning of the program.

On the one hand, these desired changes may represent nothing more than nitpicking. The transition from the conventional study program to the model curriculum took place without any revolutionary events, in part as a result of the great commitment of the instructors. The switch was instead more of a transition by way of harmonization, making it difficult for currently enrolled students to recognize the distinctions between the inherent changes to the model curriculum, the university-specific aspects, and the legal requirements which apply to all medical degree programs. On the other hand, neither instructors nor students are aware that the average study time that has traditionally been very good in Hannover [21] is still better than the average and, as a result of eliminating the first medical state examination from the HannibaL curriculum, there has been a definitive advantage in terms of time gained for students admitted from the waiting list when compared to conventional study programs and model curricula that have retained the first medical state examination [12]. For these reasons, it can be hoped that once implemented the Master Plan 2020 will also permit the trial of alternative models.

## Competing interests

The authors declare that they have no competing interests.
